# Repurposing a PICU for Adult Care in a State Mandated COVID-19 Only Hospital: Outcome Comparison to the MICU Cohort to Determine Safety and Effectiveness

**DOI:** 10.3389/fped.2021.665350

**Published:** 2021-05-14

**Authors:** Ramon E. Gist, Rohit Pinto, Niranjan Kissoon, Youssef E. Ahmed, Pia Daniel, Mitchell Hamele

**Affiliations:** ^1^Department of Pediatrics, SUNY Downstate Health Sciences University, Brooklyn, NY, United States; ^2^Department of Pediatrics and Emergency Medicine, British Columbia Children's Hospital and Sunny Hill Health Centre for Children, Child and Family Research Institute, University of British Columbia, Vancouver, BC, Canada; ^3^Department of Emergency Medicine, SUNY Downstate Health Sciences University, Brooklyn, NY, United States; ^4^Department of Pediatrics, Tripler Army Medical Center, Uniformed Service University, Bethesda, MD, United States

**Keywords:** disaster planning, surge capacity, disaster medicine, critical care, pediatric intensive care unit, critical care outcomes

## Abstract

**Objective:** The ongoing coronavirus 2019 (COVID-19) pandemic is disproportionally impacting the adult population. This study describes the experiences after repurposing a PICU and its staff for adult critical care within a state mandated COVID-19 hospital and compares the outcomes to adult patients admitted to the institution's MICU during the same period.

**Design:** A retrospective chart review was performed to analyze outcomes for the adults admitted to the PICU and MICU during the 27-day period the PICU was incorporated into the institution's adult critical care surge plan.

**Setting:** Tertiary care state University hospital.

**Patients:** Critically ill adult patients with proven or suspected COVID-19.

**Interventions:** To select the most ideal adult patients for PICU admission a tiered approach that incorporated older patients with more comorbidities at each stage was implemented.

**Measurements and Main Results:** There were 140 patients admitted to the MICU and 9 patients admitted to the PICU during this period. The mean age of the adult patients admitted to the PICU was lower (49.1 vs. 63.2 *p* = 0.017). There was no statistically significant difference in the number of comorbidities, intubation rates, days of ventilation, dialysis or LOS. Patients selected for PICU care did not have coronary artery disease, CHF, cerebrovascular disease or COPD. Mean admission Sequential Organ Failure Assessment (SOFA) score was lower in patients admitted to the PICU (4 vs. 6.4, *p* = 0.017) with similar rates of survival to discharge (66.7 vs. 44.4%, *p* = 0.64).

**Conclusion:** Outcomes for the adult patients who received care in the PICU did not appear to be worse than those who were admitted to the MICU during this time. While limited by a small sample size, this single center cohort study revealed that careful assessment of critical illness considering age and type of co-morbidities may be a safe and effective approach in determining which critically ill adult patients with known or suspected COVID-19 are the most appropriate for PICU admission in general hospitals with primary management by its physicians and nurses.

## Introduction

The ongoing coronavirus 2019 (COVID-19) pandemic, unlike previous respiratory pandemics, is disproportionally impacting adults as compared to children (1–3). Many jurisdictions throughout the United States have implemented crisis standards of care due to the tremendous surge of adults with COVID-19 requiring Intensive Care Unit (ICU) admission This is concerning because crisis standards limit the ability to provide every therapeutic option and may result in increased mortality (2, 4). Given the dire situation, attention is directed to the use of Pediatric Intensive Care Unit (PICU) beds, which comprise roughly 7.5% of all ICU beds. (5, 6) Since children represent 1–2% of COVID-19 cases, with only 0.58–2% of those requiring critical care, PICU assets may be best utilized to augment adult ICU capacity (7).

While there are guidelines on admissions, dispositions, transfers, and level of care for both pediatric (usually <18 or 21 years of age) and adult ICU settings, there is no clear evidence on when or how to care for adults in PICUs (8). However, there is an ethical imperative to offer this support if PICUs have adequate capacity to care for adult patients safely and effectively without denying these services to children. Almost half of PICU beds in the United States are in 63 large, specialized children's centers with >15 PICU beds. The remaining 281 facilities, housing 3,131 PICU beds, have <15 beds and many are in general hospitals housing both adults and children (9). The logistics in preparing a PICU for adult care will differ for general and free-standing children's hospitals. PICUs in areas enacting crisis standards of care have already started to support adult patients using a variety of novel patient care models (10–16).

On March 7, 2020, New York State Governor Andrew Cuomo declared a state of emergency due to the COVID-19 pandemic (17). To augment the state's response, the governor directed The University Hospital of Brooklyn located at The State University of New York (SUNY) Downstate Health Sciences University to become a “COVID-19 only facility” on March 28, 2020 (18). The subsequent surge in adult patients quickly overwhelmed traditional and surge critical care areas. As a result, the PICU was asked to assist in the response. This manuscript describes the experience of repurposing a PICU and its staff for adult critical care within a state mandated COVID-19 general hospital and compares the outcomes to adult patients admitted to the institution's medical ICU (MICU) during the same period.

## Methods and Materials

### Repurposing the PICU

The SUNY Downstate Health Sciences University campus is the only Academic Medical Center in Brooklyn, New York and has a 376-bed tertiary care hospital known as The University Hospital of Brooklyn. The institution has pediatric units within the adult hospital. The usual adult critical care capacity is 26 beds, with surge capacity up to 55 beds. In addition, 71 beds on the regular adult inpatient units have ventilator capability. Usual pediatric critical care capacity includes five critical care beds and seven step-down beds. Nursing staff in the adult and pediatric areas are not cross-trained and the intensivists are not dual-trained specialists.

Once the institution was designated a “COVID-19 only facility,” the pediatric emergency department was placed on diversion. To ensure continuity of care, inpatient pediatric services were diverted to local hospitals with which affiliation agreements were previously established. General and subspecialty pediatricians provided outpatient care through telemedicine. These factors caused the PICU census to dwindle, making it available to support the adult COVID-19 surge.

The initial admission triage approach was to admit any adult under the age of 30; however, this did not relieve any burden on the MICU because most patients were much older. In collaboration with the adult intensivists, the model evolved to a tiered approach that incorporated older patients with more comorbidities at each stage. Stage I allowed patients up to 40 years of age with one co-morbidity and stage 2 allowed patients up to 60 years of age with two co-morbidities. Stage 3 involved admitting any age patient regardless of comorbidities assuming other surge options had been exhausted. The hope was to allow the PICU to function with relative independence by first admitting less complex patients and gradually increasing the complexity as comfort improved and the need increased.

The pediatric intensivists served as the physicians of record and conducted morning rounds followed by brief afternoon rounds with the adult critical care consult service. Adult subspecialists performed consults as requested by the pediatric team. Nurses experienced with inpatient adult care were paired with a PICU nurse and as a team, they were assigned patients. The institution established additional code teams as well as procedure teams for intubation, prone positioning, vascular access, and chest tube placement. These teams were comprised of residents and attendings mostly from the anesthesia, surgery, and internal medicine departments. The PICU was incorporated into the institution's COVID-19 surge plan from March 31, 2020 to April 27, 2021, at which point the pediatric inpatient units were gradually decompressed and prepared to resume caring for children.

### Ethics Approval

The SUNY Downstate Health Sciences University Institutional Review Board and Privacy Board reviewed the study proposal (IRB # 1590169-1) and deemed it exempt.

### Data Analysis

To obtain study data, SUNY Downstate study team members retrospectively examined the electronic medical records (EMR) of all adult patients admitted to the PICU and MICU between March 31st and April 21st, 2020. Nasal swabs were used to obtain samples for SARS-CoV-2 testing (Hologic Panther test, Lenco Reference Lab; GeneXprt Xpress SARS-CoV-2 RNA, Cepheid). Demographics including age, sex and race were collected. Other endpoints included: ICU length of stay (LOS), ICU admission Sequential Organ Failure Assessment (SOFA) score, non-invasive and invasive mechanical ventilation requirements, ventilator days, acute dialysis requirement, chronic comorbidities present at admission, and survival to discharge. SaO_2_/FiO_2_ ratios were utilized in lieu of PaO_2_/FiO_2_ ratios when an admission blood gas was not obtained (19). Data on vasopressor/ionotropic requirements and disposition were collected for the PICU patients.

Outcomes were analyzed using the Mann–Whitney Wilcoxon test for continuous variables and the Pearson Chi-square test for categorical variables. Propensity scores were calculated using a logistic regression model. Covariate variables used for the propensity score were age and comorbidities. Standardized means were tested to ensure balance of the two patient groups after propensity score matching. All tests were two-sided, with statistical significance defined as *p* < 0.05. The tests were performed using programming language R version 4.0.1 and RStudio version 1.3.959 (Team R studio, Massachusetts, USA).

## Results

During the study period, 140 patients were admitted to the MICU and 9 patients were admitted to the PICU. All patients were above 21 years of age. The mean age of the PICU cohort was lower (49.1 vs. 63.2, *p* = 0.017). All patients in the PICU cohort were Black/African American compared to 87% in the MICU. Males were 66.7% of PICU patients and 59.5% of MICU patients. The PICU patients had a lower mean admission SOFA score (4 vs. 6.4, *p* = 0.017) (Table 1). While there was no statistically significant difference in the number of comorbidities; MICU patients had more adult-specific comorbidities (Table 2). There was no statistically significant difference in LOS, non-invasive or invasive mechanical ventilation requirement, ventilator days, or dialysis requirement. The PICU patients had a similar survival to discharge in the matched analysis (66.7 vs. 44.4%, *p* = 0.64) (Table 1).

**Table 1:** Patient demographics and outcomes in the PICU and MICU.

	**Whole cohort**			**Propensity score–matched cohort**		
**Variable**	**PICU (*n* = 9)**	**MICU (*n* = 131)**	***P*-value**	**PICU (*n* = 9)**	**MICU (*n* = 9)**	***P*-value**
Age, mean (sd)	49.1 (17.8)	63.2 (12.9)	0.019	49.1 (17.8)	49.1 (15.1)	0.944
Sex Male, *n* (%)	6 (66.7)	78 (59.5)	0.944	6 (66.7)	8 (88.9)	0.571
Race	0.86			0.076
•African American, *n* (%)	9 (100)	114 (87)		9 (100)	5 (55.6)	
•White, *n* (%)	0	8 (6.1)		0	2 (22.2)	
•Asian, *n* (%)	0	2 (1.5)		0	0	
•Hispanic, *n* (%)	0	1 (0.8)		0	0	
•Undisclosed, *n* (%)	0	6 (4.6)		0	2 (22.2)	
SOFA Score, mean (sd)	4 (2.9)	6.4 (2.9)	0.017	4 (2.9)	5.8 (2.5)	0.187
Number of Comorbidities median (IQR)	2 (1–3)	3 (2–6)	0.11	2 (1–3)	1 (1–3)	0.874
ICU LOS median	7 (5–12)	5 (3–10)	0.32	7 (5–12)	5 (4–14)	0.565
Need for non-invasive ventilation only, *n* (%)	2 (22.2)	17 (13)	0.78	2 (22.2)	4 (44.4)	0.617
Need for intubation, *n* (%)	6 (66.7)	106 (80.9)	0.38	6 (66.7)	4 (80.9)	0.635
Ventilator days median (IQR)	2 (0–6)	4 (1–8)	0.56	2 (0–6)	0 (0–3)	0.610
Dialysis, *n* (%)	2 (22.2)	30 (22.9)	1	2 (22.2)	2 (22.2)	1
Survival to discharge, *n* (%)	6(66.7)	40 (30.5)	0.06	6(66.7)	4 (44.4)	0.635

**Table 2:** Comorbidity comparison between adult patients in the PICU and MICU.

**Comorbidities**	**PICU (*n* = 9)**	**MICU (*n* = 131)**	***P*-value**
Diabetes Mellitus, *n* (%)	2 (22)	71 (54.2)	0.1304
Hypertension, *n* (%)	5 (55.6)	101 (77.1)	0.2909
Cerebrovascular Disease, *n* (%)	0	103 (78.6)	<0.001
Coronary Artery Disease, *n* (%)	0	13 (9.9)	0.69
Congestive Heart Failure, *n* (%)	0	11 (8.4)	0.79
Arrythmia, *n* (%)	1 (11.1)	18 (13.7)	1
Cerebrovascular Accident, *n* (%)	0	10 (7.6)	0.84
Chronic Obstructive Pulmonary Disease, *n* (%)	0	13 (9.9)	0.69
Other Pulmonary Dx, *n* (%)	0	20 (15.3)	0.439
Chronic Kidney Disease, *n* (%)	1 (11.1)	29 (22.1)	0.719

Five PICU patients tested positive for SARS-CoV-2. The remaining 4 were presumed positive due to the high pre-test probability during the peak of the pandemic within New York City (NYC) and the concern for false negative tests. There were no mortalities during any patents' course in the PICU; however, three (33%) died after transfer to other units or hospitals. These deaths were among the five SARS-CoV-2 positive patients, giving a case fatality rate of 60% (Table 3).

**Table 3:** Characteristics of adult patients managed in the PICU.

**Pt**	**Age and sex**	**SARS-CoV 2 PCR test results**	**Comorbidities (total)**	**SOFA score**	**PICU LOS (days)**	**Non-invasive ventilation only**	**Ventilator days**	**Dialysis**	**Disposition**	**Survival**
1	27y M	Neg	Obesity, HTN (2)	2	4	Yes	0	No	Home	Yes
2	49y F	Pos	HTN (1)	3	7	No	1	No	Home	Yes
3	26y F	Neg	Prader Willi Syndrome, ESRD, HTN (3)	9	26	No	26	Yes	Long term care	Yes
4	33y M	Neg	Type 2 DM, HTN, PE, obesity, atrial fibrillation (5)	2	5	Yes	0	No	Home	Yes
5	75y M	Pos	HTN (1)	8	12	No	6	Yes	Home	Yes
6	69y M	Pos	Type 2 DM, HTN (2)	5	11	No	6	No	Inpatient Unit	No
7	48y F	Neg	None (0)	0	2	No	0	No	Home	Yes
8	53y M	Pos	None (0)	4	12	No	9	No	MICU	No
9	62y M	Pos	Gout, obesity, OSA (3)	3	7	No	2	No	ECMO Center	No

## Discussion

There are published descriptive reports, retrospective studies, and expert opinions pertaining to the care of adults in pediatric settings during the COVID-19 pandemic (10–16). This study differs in that it describes experiences of a PICU serving as the primary team for a cohort of critically ill adult patients within a state-designated “COVID-19 only facility” and compares the outcomes to adult patients who received care in the institution's MICU during the same period.

While the PICU cohort examined here is small, some of the observed trends are consistent with ones reported in the literature. Most of the group and all patients who died were males, which is a known risk factor for ICU admission and mortality. As reported by a large adult ICU in NYC, hypertension and diabetes mellitus were the most common co-morbidities in the PICU patients (20). The case-fatality rate for the SARS-CoV-2 positive patients requiring mechanical ventilation is similar to published outcomes (53 vs. 60%). (21) This case-fatality amongst five of the nine patients who tested positive for SARS CoV-2 should be contextualized as there is a high-likelihood for false negatives given the high prevalence in the community at the time.

There were several noteworthy differences in the characteristics and outcomes between this cohort and others described in the literature. Age ≥65 and having ≥3 co-morbidities were reported to be the strongest predictors for ICU admission and mortality (20). However, within the SUNY PICU cohort, only 22% were ≥65 and only 33% had ≥3 co-morbidities. In the aforementioned report of a large ICU in NYC, 29% had coronary artery disease and 24% had congestive heart failure. Additionally, 94 and 12% of their patients required vasopressor and ionotropic support, respectively. No patient in the SUNY PICU cohort had either of these conditions nor did any require vasopressor/ionotropic support. Fewer patients in the SUNY PICU cohort required mechanical ventilation (66 vs. 93%) and hemodialysis (22.7 vs. 35.2%) (21). These differences reflect the success of the triage process in selecting less complex adult patients for admission to the PICU than are normally managed in adult ICUs.

Comparing outcomes of the SUNY Downstate PICU and MICU cohorts also demonstrates the effectiveness of the triage methodology. The low admission SOFA scores and mortality rate for the PICU cohort are matched given historical controls. However, SOFA scores alone should not be used to determine mortality risk for COVID-19 patients as the mortalities in the PICU cohort had low SOFA scores (22, 23). While the original triage approach involved an informal discussion of the patient's acuity, it did not include calculation of SOFA scores. The patients in PICU cohort also had conditions more within the scope of routine practice for a pediatric intensivist, while those with more adult-specific conditions were triaged to the MICU. The propensity scores matching analysis revealed a similar need for mechanical ventilation, length of stay and survival, which suggests that outcomes in the PICU were no worse than those in the MICU. Perhaps a formal assessment of critical illness along with age and co-morbidities (both quantity and quality) may constitute a wholistic approach in selecting the most appropriate adult patient for PICU admission during the COVID-19 surge. Developing this process was crucial in allowing the PICU team to function with autonomy, while the MICU managed the sicker patients, who would benefit from their expertise the most.

As a PICU within a general hospital where supplies and support staff needed for adult care are easily accessible, the unit was well-positioned to take on this role, perhaps more-so than a PICU within a free-standing children's hospital. Respiratory therapists from the adult services were available to support the pediatric respiratory therapists. Invasive and non-invasive ventilators in the PICU had pediatric and adult capabilities. The EMR had the capacity to order any medication needed due to the existence of an adult formulary. The pharmacy staff was accustomed to adult dosing, preparation, medication interactions, and safety profiles. The hospital laboratories were set up for adult tests with integration of critical values within the EMR. The radiology department has adult screening protocols for imaging and interventional procedures. The central sterile processing department was able to provide all necessary equipment for adult care. Implementing procedure and additional code teams anecdotally relieved the anxiety and apprehension amongst the pediatric team members.

While there were no institution-specific steps taken for medicolegal protections, on April 7, 2020 New York State enacted the Emergency or Disaster Treatment Protection Act, which provided retroactive and ongoing immunity to healthcare facilities and providers treating patients with known or suspected COVID-19 (24).

Our study had several limitations. This is an observational study with a small number of patients admitted to a single PICU, which limits the study's power and the ability to draw generalizable conclusions. Despite the similar outcomes in the matched cohorts, these results should be interpreted with understanding of the biases that arise from balancing cohorts. The conclusions reached in this study should be further investigated with a larger number of patients.

## Conclusions

PICUs can support mass critical care during the COVID-19 pandemic once the safety and care of critically ill pediatric patients is first ensured through regional coordination and partnerships with near-by facilities. Regional delineation of care based on epidemiologic nuances may facilitate efficient care delivery when intensive care becomes a limited resource. The ideal institutional system-based approach should include logistic support, training on adult treatment protocols, formal consultative relationships, and medico-legal protections for staff and facilities. This single center cohort study revealed that careful assessment of critical illness considering age and type of co-morbidities may be a safe and effective approach in determining which critically ill adult patients with known or suspected COVID-19 are the most appropriate for PICU admission in general hospitals with primary management by its physicians and nurses. Outcomes for the adult patients who received care in the PICU did not appear to be worse than those who were admitted to the MICU during this time.

## Data Availability Statement

The raw data supporting the conclusions of this article will be made available by the authors, without undue reservation.

## Ethics Statement

The studies involving human participants were reviewed and approved by The SUNY Downstate Health Sciences University Institutional Review Board and Privacy Board reviewed the study proposal (IRB # 1590169-1) and deemed it exempt. Written informed consent for participation was not required for this study in accordance with the national legislation and the institutional requirements.

## Disclosure

LTC Hamele is an officer in the United States Army.

## Author Contributions

RG led study design and is the primary author of the manuscript. RP led data collection efforts and contributed to the methods and results sections. YA led statistical analysis, assisted with data collection, and contributed to the methods and results section. PD led Institutional Review Board application preparation and editing of the document. MH and NK were involved in framing the manuscript in the larger context of the COVID-19 pandemic. All authors provided critical feedback and helped shape the research, analysis, and manuscript.

## Conflict of Interest

The authors declare that the research was conducted in the absence of any commercial or financial relationships that could be construed as a potential conflict of interest.
